# Changes in Hospitalization and Infection Burden in Patients with Multiple Myeloma Before and During the COVID-19 Pandemic

**DOI:** 10.3390/jcm15124613

**Published:** 2026-06-14

**Authors:** Sunil Lakhwani, Cristian L. Gutiérrez-Padilla, Raúl Domínguez-Guerra, Andrea R. Rodríguez-Suárez, Marta Díaz-López, Alejandro Martín-Martín, Miguel T. Hernández-García

**Affiliations:** 1 Department of Internal Medicine, Dermatology and Psychiatry, Universidad de La Laguna, 38200 San Cristobal de La Laguna, Islas Canarias, Spain; clupad1773@gmail.com (C.L.G.-P.); rauldguezguerra@gmail.com (R.D.-G.); arodsuak@gobiernodecanarias.org (A.R.R.-S.); mthernan@ull.edu.es (M.T.H.-G.); 2 Department of Hematology, Hospital Universitario de Canarias, 38320 San Cristobal de La Laguna, Islas Canarias, Spain; mdialopu@gmail.com (M.D.-L.); alemartinmartin@hotmail.es (A.M.-M.)

**Keywords:** multiple myeloma, hospitalization, healthcare utilization, infection, respiratory infections, nosocomial pneumonia, COVID-19, epidemiolo

## Abstract

**Background:** The increasing incidence and improved survival rates of multiple myeloma (MM) have led to a growing healthcare burden, particularly in terms of hospitalizations. In addition, infection-related complications remain a major cause of morbidity and mortality in these patients. The impact of infection control measures implemented during the COVID-19 pandemic on hospitalization patterns in MM is not well-established. **Methods:** We conducted a retrospective observational study including all hospital admissions of patients with MM in a tertiary hospital in Spain across three different periods: 2008, 2018, and May 2020 to April 2021 (COVID-19 pandemic period). We analyzed the proportion of admissions, cumulative length of stay, causes of hospitalization, and infection-related complications. **Results:** The proportion of hospitalizations due to MM increased significantly from 14% in 2008 to 29.8% in 2018 (*p* < 0.001), along with a parallel increase in cumulative length of stay (14.4% vs. 27.1%, *p* < 0.001). During the COVID-19 period, a significant reduction in the proportion of admissions was observed compared to 2018 (21.2% vs. 29.8%, *p* = 0.0029), while cumulative length of stay showed a non-significant decrease. The proportion of infection-related admissions remained stable during the pandemic period, although the absolute number of infections decreased, including respiratory infections. Notably, the incidence of nosocomial pneumonia decreased significantly (26.3% vs. 9.6%, *p* = 0.028). **Conclusions:** Compared with 2008, patients with MM accounted for a substantially higher proportion of hospital admissions and cumulative hospital stay in 2018, reflecting the increasing healthcare burden associated with this disease. During the COVID-19 period, a significant reduction in nosocomial pneumonia was observed, suggesting that infection-control strategies may help reduce respiratory complications in patients with MM.

## 1. Introduction

Multiple myeloma (MM) is the second most common hematologic malignancy after non-Hodgkin lymphoma [1,2]. Its incidence ranges from 4.5 to 6 cases per 100,000 inhabitants per year [2,3], although higher incidence rates (until 8 to 10 cases per 100,000 inhabitants per year) have been reported in certain regions, including areas of Spain such as the Canary Islands [4]. MM is more frequent in males and primarily affects older individuals, with a median age at diagnosis of approximately 70 years [2,3]. Population aging, among other factors, has contributed to a progressive increase in its incidence, and this trend is expected to continue in the coming decades [5].

In addition, the introduction of novel therapeutic agents over the past two decades has led to a substantial improvement in survival [6,7]. As a consequence of both increasing incidence and improved survival, the prevalence of MM has risen substantially, resulting in a growing healthcare burden.

The impact of MM on healthcare systems extends beyond disease-specific treatment. Patients frequently require medical attention for disease-related complications, treatment administration, supportive care, and infectious events. Consequently, healthcare utilization among patients with MM has increased substantially in recent years. Previous studies have demonstrated a marked rise in outpatient activity and healthcare expenditure associated with MM, reflecting the growing number of patients living with the disease and receiving prolonged treatment [8,9,10]. However, comparatively less information is available regarding changes in hospitalization patterns, despite hospital admissions remaining one of the major contributors to healthcare resource utilization in this population.

Infections remain one of the leading causes of morbidity and mortality in patients with MM and account for a significant proportion of hospital admissions [11,12,13]. The increased susceptibility to infection is multifactorial and results from disease-related immune dysfunction, advanced age, comorbidities, and treatment-induced immunosuppression [2,3]. Patients are particularly vulnerable during the first months after diagnosis and during periods of intensive treatment. Respiratory infections are particularly frequent and are associated with considerable clinical impact. Preventive strategies such as vaccination, antimicrobial prophylaxis, and infection-control measures have therefore become increasingly important components of MM management [2,3].

The COVID-19 pandemic represented an unprecedented public health event that led to the widespread implementation of infection-control measures, including mask use, social distancing, visitor restrictions, and enhanced hygiene practices. Although these interventions were primarily intended to reduce SARS-CoV-2 transmission, they may also have influenced the incidence of other respiratory infections, particularly among immunocompromised patients. Nevertheless, the impact of these measures on hospitalization patterns and infection-related complications in patients with MM remains poorly characterized.

Despite the increasing prevalence of MM and the recognized impact of infections on patient outcomes, relatively few studies have evaluated how hospitalization patterns have evolved over time or how infection-control measures may have influenced inpatient complications in this population.

In this context, we aimed to assess whether the increasing prevalence of MM has been associated with changes in hospitalization burden. In addition, we sought to evaluate the impact of COVID-19-related infection-control measures on hospitalization patterns and infection-related complications in patients with MM. Understanding these changes is important to optimize healthcare resource utilization and to identify potential strategies for reducing preventable complications in this vulnerable population.

## 2. Methods

### 2.1. Study Design and Setting

We conducted a retrospective observational study analyzing hospital admissions in the Department of Hematology of a tertiary care hospital in Tenerife, Canary Islands, Spain. Three different time periods were evaluated:

- 1 January to 31 December 2008 (baseline period);
- 1 January to 31 December 2018 (pre-COVID period);
- 1 May 2020 to 30 April 2021 (COVID-19 pandemic period).

These periods were selected to represent three distinct clinical scenarios: a baseline period before the widespread use of novel anti-myeloma therapies (2008), a contemporary pre-pandemic period reflecting current clinical practice (2018), and a period characterized by the implementation of COVID-19-related infection control measures (May 2020–April 2021).

Intermediate years were not included because the aim of the study was not to perform a year-by-year trend analysis but rather to compare representative periods before and during major epidemiological and organizational changes affecting the management of multiple myeloma.

### 2.2. Study Population

We included all hospital admissions of patients diagnosed with multiple myeloma (MM) according to the diagnostic criteria in use at each study period [14,15]. Patients with plasma cell leukemia were also included in the MM group. Admissions corresponding to other hematologic diseases were also recorded and classified into the following categories:

- Acute leukemia (AL);
- Myelodysplastic syndromes and chronic myelomonocytic leukemia (MDS/CMML);
- Chronic myeloproliferative neoplasms (MPNs);
- Lymphoproliferative disorders (LPDs);
- Autoimmune hematologic diseases (AHDs);
- Other monoclonal gammopathies (including AL amyloidosis and solitary plasmacytoma; other MGs).

### 2.3. Data Collection

For patients with MM, we collected demographic variables (age and sex), disease-related characteristics at diagnosis, including stage and laboratory parameters when available, and variables related to hospitalization episodes. These included cause of admission, length of hospital stay, infectious complications, and nosocomial events. Data were extracted from electronic medical records and entered into a predefined encoded database for analysis.

For infection-related admissions, additional information regarding the type of infection and site of involvement was collected whenever available. Respiratory infections were analyzed separately because they represent one of the most frequent infectious complications in patients with MM. Nosocomial complications were identified from hospitalization records and classified according to their clinical presentation.

### 2.4. Study Objectives and Comparisons

To assess changes in hospitalization patterns over time, we compared the baseline period (2008) with the pre-COVID period (2018). To evaluate the impact of COVID-19-related preventive measures, we compared the pre-COVID period (2018) with the pandemic period (May 2020–April 2021). The main variables analyzed included the proportion of hospital admissions due to MM, cumulative length of hospital stay, causes of admission, and nosocomial complications of these patients. In addition to analyzing MM admissions, the distribution of admissions and cumulative length of stay for other hematologic diseases was also recorded to provide context regarding the overall activity of the hematology department during each study period.

### 2.5. Statistical Analysis

Descriptive statistics were used to summarize the study variables. Categorical variables were expressed as absolute frequencies and percentages. Comparisons between study periods were performed using the chi-square test or Fisher’s exact test when appropriate. The analysis focused on differences in the proportion of admissions, causes of hospitalization, infection-related complications, and cumulative hospital stay across the selected periods. All statistical analyses were performed using IBM SPSS software for Windows (version 23.0). All tests were two-sided, and a *p*-value < 0.05 was considered statistically significant.

Given the exploratory nature of the study, no formal sample size calculation was performed. All available admissions during the predefined study periods were included in the analysis.

### 2.6. Ethical Considerations

The study was conducted in accordance with the principles of the Declaration of Helsinki and was approved by the local Research Ethics Committee. The requirement for informed consent was waived due to the retrospective nature of the study. Patient confidentiality was maintained throughout the study. Data were anonymized prior to analysis and handled in accordance with applicable data protection regulations.

## 3. Results

A total of 408 hospital admissions were recorded in 2008, 531 in 2018, and 411 between May 2020 and April 2021. Of these, 57 admissions (14%) in 2008 corresponded to patients with multiple myeloma (MM), compared to 158 admissions (29.8%) in 2018, representing a significant increase (*p* < 0.001). During the COVID-19 period, 87 admissions (21.2%) were attributed to MM, reflecting a significant decrease compared to 2018 (*p* = 0.0029). The full distribution of admissions by hematologic disease is shown in Table 1.

Regarding cumulative length of stay, MM accounted for 665 days out of 4612 total hospital days (14.4%) in 2008, compared to 1982 days out of 7307 (27.1%) in 2018, a statistically significant increase (*p* < 0.001). During the pandemic period, MM accounted for 1370 out of 5217 total hospital days (26.3%), with no significant difference compared to 2018 (*p* = 0.2814). Detailed data on cumulative length of stay are provided in Table 2.

The causes of admission are summarized in Table 3. In 2008, 17 of 57 admissions (29.8%) were due to infections, 12 (21%) were for treatment administration, 10 (17.5%) were related to disease onset, and 4 (7%) were for autologous stem cell transplantation. In 2018, the proportion of admissions for treatment increased to 59 of 158 (37.3%), while 31 admissions (19.6%) were due to infections, 11 (7%) to disease onset, and 11 (7%) to autologous stem cell transplantation. Compared with 2008, the increase in treatment-related admissions and the decrease in admissions due to disease onset were statistically significant (*p* = 0.025 and *p* = 0.021, respectively).

During the COVID-19 period, 17 of 87 admissions (19.5%) were due to infections, 15 (17.2%) to treatment, 16 (18.4%) to disease onset, and 17 (19.5%) to autologous stem cell transplantation. Compared with 2018, there were no significant differences in infection-related admissions. However, the proportion of admissions for treatment decreased significantly (*p* = 0.001), while admissions due to disease onset (*p* = 0.006) and autologous stem cell transplantation (*p* = 0.003) increased significantly.

Among the 31 infection-related admissions in 2018, 18 (58.1%) were due to respiratory infections, while 13 were due to other causes. During the pandemic period, 8 of 17 infection-related admissions (47.1%) were respiratory, and 9 were due to other causes. Despite a reduction in absolute numbers, no statistically significant differences were observed in the proportion of respiratory infections (*p* = 0.464).

When analyzing nosocomial complications, 57 events were recorded in 2018, including 15 cases of nosocomial pneumonia (26.3%). During the COVID-19 period, 52 nosocomial complications (events) were observed, of which only 5 corresponded to nosocomial pneumonia (9.6%), representing a statistically significant reduction (*p* = 0.028). Detailed data on nosocomial complications are presented in Table 4.

## 4. Discussion

In this study, we observed a significant increase in hospitalizations among patients with multiple myeloma (MM) between 2008 and 2018, accompanied by a parallel rise in the proportion of cumulative hospital stay. These findings probably reflect an increasing healthcare burden associated with MM, likely driven by increased incidence and improved survival due to advances in treatment.

The observed increase in the proportion of MM admissions between 2008 and 2018 is likely multifactorial. Population aging may have contributed to a higher incidence of MM, while advances in therapy have substantially improved survival. As a result, a growing number of patients are living with MM for prolonged periods and remain exposed to disease-related complications, treatment-related adverse events, and the need for supportive care. Consequently, the increasing prevalence of MM is expected to generate a progressively greater demand for healthcare resources, including hospital admissions, outpatient visits, and specialized treatments.

Our findings are consistent with previous reports showing substantial healthcare resource utilization and costs associated with MM. Previous studies have demonstrated that changes in treatment patterns and improved survival have been accompanied by increasing healthcare demands, while unplanned admissions continue to generate a significant economic burden [8,10].

The evolution of MM from a rapidly fatal malignancy to a chronic disease has profoundly changed its healthcare implications. Historically, many patients experienced relatively short survival and limited treatment options. In contrast, contemporary patients frequently receive multiple lines of therapy over several years, including immunomodulatory agents, proteasome inhibitors, monoclonal antibodies, cellular therapies, and bispecific antibodies. Although these advances have substantially improved outcomes, they have also increased the complexity of patient management. Long-term monitoring, treatment-related toxicities, supportive care needs, and management of infectious complications have become increasingly important components of routine clinical practice. Consequently, healthcare systems must adapt to the growing demands associated with an expanding population of patients living with MM.

The main change in the reasons for hospitalization over time was the increase in admissions for treatment administration. This finding may be explained by the greater availability of novel therapies and by organizational factors within the healthcare system, such as the need for inpatient monitoring during the first administration of certain drugs (e.g., daratumumab). We have not found any studies that directly analyze these differences, although an increase in hospitalizations has been described over the years [10].

During the COVID-19 pandemic period, we observed a significant reduction in the number of hospital admissions among MM patients compared with 2018, although the decrease in cumulative length of stay did not reach statistical significance. This reduction may be partly explained by changes in clinical practice, including the shift of certain treatments, such as daratumumab, to the outpatient setting. In addition, healthcare reorganization during the COVID-19 pandemic, fluctuations in hospital capacity, and efforts to minimize inpatient exposure may also have contributed to the observed changes in hospitalization patterns.

An additional finding was the increase in admissions related to disease onset during the COVID-19 period. Several factors may have contributed to this observation, including the rising incidence of MM and possible delays in diagnosis during the pandemic. Despite major therapeutic advances, many patients continue to be diagnosed only after developing clinically significant end-organ damage, such as pathological fractures, severe anemia, or renal impairment. These findings highlight the persistent challenge of achieving earlier diagnosis in MM. Greater awareness of myeloma-related symptoms among healthcare professionals, together with strategies aimed at facilitating earlier detection, may help reduce the burden of advanced disease at presentation and potentially prevent some hospitalization episodes.

Regarding infections, the proportion of infection-related admissions remained essentially unchanged between 2018 and the pandemic period. However, the absolute number of infection-related admissions decreased in parallel with the reduction in overall MM admissions. In addition, we observed a reduction in the absolute number of respiratory infections and, more importantly, a significant decrease in nosocomial pneumonia. This finding is particularly relevant because infections remain one of the leading causes of morbidity and mortality in patients with MM. The observed reduction in nosocomial pneumonia may have been influenced by multiple factors, including mask use, enhanced infection-control policies, visitor restrictions, social distancing, changes in healthcare-seeking behavior, and broader societal measures implemented during the COVID-19 pandemic. Therefore, the observed reduction in nosocomial pneumonia should probably be interpreted as the result of a combination of infection-control interventions rather than any single preventive measure.

Infection-control measures implemented during the pandemic may have contributed to reducing droplet transmission, which could plausibly decrease the risk of respiratory infections, including nosocomial pneumonia. However, prospective studies are needed to determine which interventions provide the greatest benefit.

Respiratory infections are among the most clinically relevant complications in MM, causing significant morbidity and mortality. Several studies have identified infections as a major contributor to mortality in MM, particularly during periods of active treatment [11,12,13,16,17]. Therefore, even modest reductions in respiratory infectious complications may translate into meaningful clinical benefits. Although our study was not designed to evaluate patient survival or treatment outcomes, the observed reduction in nosocomial pneumonia suggests that preventive strategies targeting respiratory pathogen transmission deserve further investigation in this population.

The reduction in nosocomial pneumonia deserves particular attention, because hospital-acquired respiratory infections are associated with considerable morbidity in patients with MM. These infections frequently prolong hospital stay, increase the need for antimicrobial therapy, and may delay the administration of anti-myeloma treatment. Although causality cannot be established in the present study, the significant reduction observed during the COVID-19 period suggests that selected infection-control interventions may have a measurable impact on clinically relevant complications among hospitalized patients with MM.

Interestingly, hospitalization patterns were not uniform across hematologic diseases. For example, admissions for acute leukemia increased substantially during the pandemic period, suggesting that disease-specific factors and organizational priorities may have influenced hospitalization patterns differently across diagnostic groups. Therefore, the findings observed in MM should not be extrapolated to other hematologic diseases.

In recent years, the introduction of CAR-T cell therapy and bispecific antibodies has significantly improved outcomes in MM [18]. However, infections remain a common and clinically relevant complication associated with these treatments [19]. In this context, the implementation of simple and low-cost preventive measures could be particularly valuable in reducing infection-related morbidity in these high-risk patients. Our findings may have implications beyond the COVID-19 pandemic. Identifying simple and sustainable measures capable of reducing hospital-acquired respiratory infections could be particularly relevant in the current era of increasingly immunosuppressive therapies. Future studies should evaluate whether selected infection-control interventions may provide benefits in high-risk MM populations outside pandemic settings.

This study has several limitations that should be acknowledged. First, its retrospective and single-center design may limit the generalizability of the findings. Second, only three representative periods were analyzed; therefore, our results should not be interpreted as evidence of a continuous temporal trend. Third, changes in clinical practice and hospital organization over time may have influenced hospitalization patterns independently of disease-related factors. These include the introduction of novel therapies, expansion of outpatient treatment programs, fluctuations in hospital capacity, and healthcare reorganization during the COVID-19 pandemic. Finally, the study was not specifically designed to evaluate the impact of individual infection-control measures, and therefore causality between these interventions and the observed reduction in nosocomial pneumonia cannot be established.

Additional limitations should also be considered. The study evaluated hospitalization episodes rather than individual patients; therefore, repeated admissions by the same patient may have influenced some of the observed patterns. Furthermore, detailed information regarding disease status, treatment exposure, comorbidities, vaccination status, and severity of infectious complications was not consistently available for all study periods and could not be incorporated into the analysis. Finally, because this was an observational study conducted in routine clinical practice, unmeasured confounding factors may have contributed to the differences observed between study periods.

From a practical perspective, our findings highlight the importance of continuously evaluating hospitalization patterns in MM as therapeutic strategies evolve. Hospital admissions remain a major component of healthcare utilization in this disease and represent a substantial burden for patients, caregivers, and healthcare systems. Understanding the factors associated with hospitalization may facilitate the development of preventive strategies and help optimize resource allocation. In addition, the potential reduction in nosocomial respiratory complications observed during the pandemic period raises the possibility that selected infection-control measures could remain beneficial in specific high-risk settings beyond the COVID-19 era.

## 5. Conclusions

Compared with 2008, patients with MM accounted for a substantially higher proportion of hospital admissions and cumulative hospital stay in 2018, reflecting the increasing healthcare burden associated with this disease. During the COVID-19 period, a significant reduction in nosocomial pneumonia was observed, supporting further evaluation of infection-control strategies to reduce respiratory complications in patients with MM.

## Figures and Tables

**Table 1:** Distribution and comparison of hospital admissions by hematologic disease across the different study periods.

Admission Episodes						
	2008 (n = 408)	2018 (n = 531)	*p*-Value	2018 (n = 531)	2020–21 (n = 411)	*p*-Value
**MM**	57 (14%)	158 (29.8%)	*p < 0.001*	158 (29.8%)	87 (21.2%)	*p = 0.0029*
**AL**	127 (31.1%)	60 (11.3%)	*p < 0.001*	60 (11.3%)	118 (28.7%)	*p < 0.001*
**MDS/CMML**	24 (5.9%)	33 (6.2%)	NS	33 (6.2%)	22 (5.3%)	NS
**MPN**	12 (2.9%)	11 (2.1%)	NS	11 (2.1%)	3 (0.7%)	NS
**LPD**	155 (38%)	214 (40.3%)	NS	214 (40.3%)	154 (37.5%)	NS
**AHD**	13 (3.2%)	27 (5.1%)	NS	27 (5.1%)	11 (2.7%)	NS
**Other MG**	12 (2.9%)	14 (2.6%)	NS	14 (2.6%)	5 (1.2%)	NS
**Others**	8 (2%)	14 (2.6%)	NS	14 (2.6%)	11 (2.7%)	NS

Abbreviations: MM: multiple myeloma; AL: acute leukemia; MDS: myelodysplastic syndromes; CMML: chronic myelomonocytic leukemia; MPN: myeloproliferative neoplasms; LPD: lymphoproliferative disorders; AHD: autoimmune hematologic diseases; MG: monoclonal gammopathies; NS: not significant. Values in italics indicate statistically significant differences.

**Table 2:** Distribution and comparison of cumulative length of hospital stay by hematologic disease across the different study periods.

Accumulated Hospital Stays						
	2008 (n = 4612)	2018 (n = 7307)	*p*-Value	2018 (n = 7307)	2020–21 (n = 5217)	*p*-Value
**MM**	665 (14.4%)	1982 (27.1%)	*p < 0.001*	1982 (27.1%)	1370 (26.3%)	NS
**AL**	1655 (35.9%)	1406 (19.2%)	*p < 0.001*	1406 (19.2%)	1660 (31.8%)	*p < 0.001*
**MDS/CMML**	187 (4.1%)	673 (9.2%)	*p < 0.001*	673 (9.2%)	289 (5.5%)	*p < 0.001*
**MPN**	141 (3.1%)	150 (2.1%)	*p = 0.0054*	150 (2.1%)	14 (0.3%)	*p < 0.001*
**LPD**	1745 (37.8%)	2486 (34%)	*p < 0.001*	2486 (34%)	1502 (28.8%)	*p < 0.001*
**AHD**	42 (0.9%)	284 (3.9%)	*p < 0.001*	284 (3.9%)	111 (2.1%)	*p < 0.001*
**Other MG**	90 (1.9%)	152 (2.1%)	NS	152 (2.1%)	134 (2.6%)	NS
**Others**	87 (1.9%)	174 (2.4%)	NS	174 (2.4%)	137 (2.6%)	NS

Abbreviations: MM: multiple myeloma; AL: acute leukemia; MDS: myelodysplastic syndromes; CMML: chronic myelomonocytic leukemia; MPN: myeloproliferative neoplasms; LPD: lymphoproliferative disorders; AHD: autoimmune hematologic diseases; MG: monoclonal gammopathies; NS: not significant. Values in italics indicate statistically significant differences.

**Table 3:** Distribution and comparison of causes of hospital admissions in patients with multiple myeloma across the different study periods.

Cause of Admission						
	2008 (n = 57)	2018 (n = 158)	*p*-Value	2018 (n = 158)	2020–21 (n = 87)	*p*-Value
**Infection**	17 (29.8%)	31 (19.6%)	NS	31 (19.6%)	17 (19.5%)	NS
**Treatment**	12 (21%)	59 (37.3%)	*p = 0.0249*	59 (37.3%)	15 (17.2%)	*p = 0.0010*
**Disease onset**	10 (17.5%)	11 (7%)	*p = 0.0211*	11 (7%)	16 (18.4%)	*p = 0.0062*
**ASCT**	4 (7%)	11 (7%)	NS	11 (7%)	17 (19.5%)	*p = 0.0031*
**Progression**	1 (1.7%)	8 (5.1%)	NS	8 (5.1%)	7 (8%)	NS
**Renal failure**	0 (0%)	5 (3.2%)	NS	5 (3.2%)	1 (1.1%)	NS
**Hypercalcemia**	0 (0%)	1 (0.6%)	NS	1 (0.6%)	1 (1.1%)	NS
**Skeletal event**	1 (1.7%)	5 (3.2%)	NS	5 (3.2%)	1 (1.1%)	NS
**Others**	13 (22.8%)	27 (17%)	NS	27 (17%)	12 (13.8%)	NS

Abbreviations: ASCT: autologous stem cell transplantation; NS: not significant. Values in italics indicate statistically significant differences.

**Table 4:** Distribution and comparison of nosocomial complications in patients with multiple myeloma during the pre-pandemic and COVID-19 periods.

Nosocomial Complication	2018 (n = 57 Events)	2020–21 (n = 52 Events)	*p*-Value
**Nosocomial pneumonia**	15 (26.3%)	5 (9.6%)	*p = 0.028*
**Urinary tract infection**	6 (10.5%)	7 (13.5%)	NS
**Catheter related infection**	4 (7.0%)	4 (7.7%)	NS
**Other infections**	8 (14.0%)	9 (17.3%)	NS
**Acute kidney injury**	6 (10.5%)	8 (15.4%)	NS
**Venous thromboembolism**	2 (3.5%)	2 (3.8%)	NS
**Acute confusional syndrome**	8 (14.0%)	7 (13.5%)	NS
**Other**	8 (14.0%)	10 (19.2%)	NS

Abbreviations: NS: not significant. Values in italics indicate statistically significant differences.

## Data Availability

The data presented in this study are available from the corresponding author upon reasonable request. The data are not publicly available due to privacy and ethical restrictions.
